# Metagenomic analysis of carbohydrate-active enzymes and their contribution to marine sediment biodiversity

**DOI:** 10.1007/s11274-024-03884-5

**Published:** 2024-02-13

**Authors:** Rafael López-Sánchez, Eria A. Rebollar, Rosa María Gutiérrez-Ríos, Alejandro Garciarrubio, Katy Juarez, Lorenzo Segovia

**Affiliations:** 1 Departamento de Ingeniería Celular y Biocatálisis, Instituto de Biotecnología, Universidad Nacional Autónoma de México, Cuernavaca, Morelos Mexico; 2 Centro de Ciencias Genómicas, Universidad Nacional Autónoma de México, Cuernavaca, Morelos Mexico; 3 Departamento de Microbiología Molecular, Instituto de Biotecnología, Universidad Nacional Autónoma de México, Cuernavaca, Morelos Mexico

**Keywords:** Anoxic, Bioinformatics, CAZymes, Oxic, Marine sediments, Metagenomics

## Abstract

Marine sediments constitute the world’s most substantial long-term carbon repository. The microorganisms dwelling in these sediments mediate the transformation of fixed oceanic carbon, but their contribution to the carbon cycle is not fully understood. Previous culture-independent investigations into sedimentary microorganisms have underscored the significance of carbohydrates in the carbon cycle. In this study, we employ a metagenomic methodology to investigate the distribution and abundance of carbohydrate-active enzymes (CAZymes) in 37 marine sediments sites. These sediments exhibit varying oxygen availability and were isolated in diverse regions worldwide. Our comparative analysis is based on the metabolic potential for oxygen utilisation, derived from genes present in both oxic and anoxic environments. We found that extracellular CAZyme modules targeting the degradation of plant and algal detritus, necromass, and host glycans were abundant across all metagenomic samples. The analysis of these results indicates that the oxic/anoxic conditions not only influence the taxonomic composition of the microbial communities, but also affect the occurrence of CAZyme modules involved in the transformation of necromass, algae and plant detritus. To gain insight into the sediment microbial taxa, we reconstructed metagenome assembled genomes (MAG) and examined the presence of primary extracellular carbohydrate active enzyme (CAZyme) modules. Our findings reveal that the primary CAZyme modules and the CAZyme gene clusters discovered in our metagenomes were prevalent in the Bacteroidia, Gammaproteobacteria, and Alphaproteobacteria classes. We compared those MAGs to organisms from the same taxonomic classes found in soil, and we found that they were similar in its CAZyme repertoire, but the soil MAG contained a more abundant and diverse CAZyme content. Furthermore, the data indicate that abundant classes in our metagenomic samples, namely Alphaproteobacteria, Bacteroidia and Gammaproteobacteria, play a pivotal role in carbohydrate transformation within the initial few metres of the sediments.

**Supplementary Information:**

The online version contains supplementary material available at 10.1007/s11274-024-03884-5.

## Introduction

The ocean floor is the recipient of all the organic matter coming from the water column and is considered the major carbon repository on the planet. Therefore, microorganisms that live in marine sediments control the storage of massive amounts of carbon (Orcutt et al. 2011). These microbes that live on and below the sea floor represent more than 10^29^ cells living, a number roughly equal to the number of microorganisms in seawater and soil (Kallmeyer et al. 2012).

Marine sediments can be classified, depending on the availability of electron acceptors such as oxygen and sulphur, into oxic or anoxic. In the oxic subseafloor, the penetration of O_2_ and the resulting limitation of the electron donor result in a unique community structure, compared to anoxic sediments (Orsi 2018). In regions of the seabed with subseafloor anoxia, oxygen is typically consumed in the upper centimetres of the sediment below (Froelich et al. 1979; D’Hondt et al. 2004).

In both types of marine sediments (oxic and anoxic), microbial communities process both organic and inorganic carbon and contribute to the cycling of nutrients such as sulphur, nitrogen, and iron (Parkes et al. 2014). Despite the global importance of these organisms, marine sediments are among the least understood environments. This is in part due to the difficulty of sampling, especially in the deep sea, and to the complexity of their inhabiting communities. However, recent examination of prokaryote genes, transcripts, and metagenomes has highlighted the importance of polysaccharides and their transformations for carbon metabolism in the ocean (Teeling et al. 2012, 2016). Therefore, a closer examination of the marine polysaccharide cycle and the communities driving their degradation is necessary. Although polysaccharides constitute a large fraction of phytoplankton and macroalgae bodies (Biersmith and Benner 1998) as well as dissolved and particulate organic matter (DOM and POM, respectively) (Lee et al. 2001), little is known about their biogeochemical processing compared to other major compound classes, such as proteins, lipids, and nucleic acids. Carbohydrate-active enzymes (CAZymes) are proteins with known activities involved in the synthesis and degradation of glycoconjugates, oligo- and polysaccharides. They typically correspond to 1–3% of the genes of a living organism (Cantarel et al. 2009). These enzymes play essential roles in life not only as structure and energy reserve components but also in many intracellular and intercellular recognition events. CAZymes are often involved in immune and host–pathogen interactions and are involved in human and agricultural-related diseases. CAZymes have been classified and annotated in the CAZy database since 1998. This is a specialist database dedicated to the display and analysis of genomic, structural, and biochemical information on carbohydrate-activated enzymes (CAZymes) (Lombard et al. 2013).

Here, we present a comparative study of carbohydrate active enzymes (CAZymes) from sediment metagenomes from different locations in the world to better understand their role in the storage or degradation of carbohydrates and derivatives.

## Methods

### Selection of metagenomic data

**Fig. 1:**
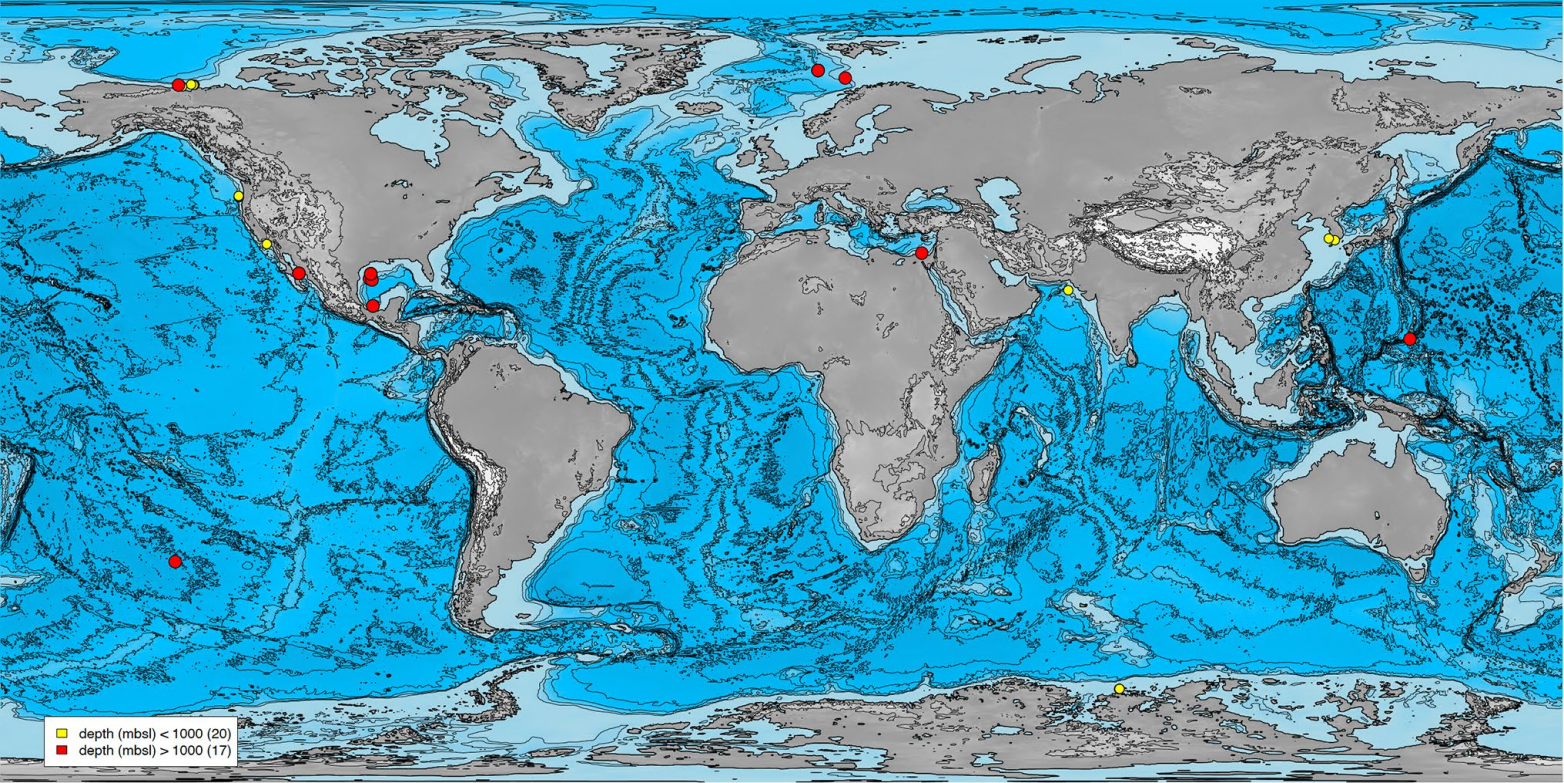
Map with the location of metagenome samples using marmap package from R (Pante and Simon-Bouhet 2013). Colour scales indicate depth in metres below sea level. The numbers in parentheses indicate the number of metagenomes

Upon identification of suitable BioProjects, the metagenome shotgun sequences were downloaded from the NCBI database. The raw data recovered from 37 metagenome samples from 12 BioProjects representing marine samples included valuable metadata associated with each sample, including latitude/longitude coordinates, metres below the sea floor, and metres below sea level (National Center for Biotechnology Information 2019). This additional information provided an important context for understanding the spatial distribution and environmental characteristics of the marine ecosystems sampled. Sediment samples were taken from all over the world with a depth range of 0–7942 m below sea level (mbsl) and 0 to 2.23 m below the sediment floor (mbsf) (Fig. 1). To reduce bias from sequencing, samples not sequenced by Illumina were discarded (Supplementary Table 1).

### Quality control and pre-processing

Quality control procedures were executed using widely used tools and software, such as Trimmomatic (Bolger et al. 2014) and FastQC (Andrews 2010). All reads from samples that did not pass the QC filters (read quality >  = Q20) were discarded.

### Taxonomic analysis of metagenomic reads

For taxonomic analysis of the reads, we used the Kraken2 specific database based on k-mer spectra from complete RefSeq genomes and the NCBI nt database (downloaded 7/09/2021) (Wood et al. 2019). The annotation tables were formatted for the R ggplot2 library to generate stacked bar plots at different taxonomic levels. The integrated matrices obtained for the 37 samples were written using R, bash, Perl, and Python and are available at https://github.com/jenniferlu717/KrakenTools.

### Metagenomic sequence read assembly and functional analysis of the metagenome

A de novo assembly was made for each sample using MEGAHIT v1.1.1–2 with the parameter ‘metasensitive’ recommended for diverse samples (Li et al. 2015). The ORFs of each sample were predicted using the Prodigal-v2.6.3 tool (Hyatt et al. 2010). Samples with less than one million genes were discarded from this study. We used HMMER-3.2.1 (Eddy 2011) (hmmscan cutoffs: E-value < 1e-15, coverage > 0.35) to annotate CAZymes against the HMM database V9 of dbCAN2 (Zhang et al. 2018). The substrate specificities of CAZymes were inferred by manual inspection of CAZy (Lombard et al. 2013, 2014). Extracellular CAZymes were annotated using SignalP V-5.0 (Almagro et al. 2019). Heme-copper oxygen reductases (HCO) and nitric oxide reductases (NOR) were analysed using Diamond (parameters “ultra-sensitive”) against the HCO database (Sousa et al. 2011). Normalisation of gene counts between CAZymes samples and Heme-copper oxygen reductase genes was carried out using the equation: (Number of genes annotated/Number of total genes in sample) × 10^6^.

### Diversity and statistical analysis

Statistical analyses were performed using R-v. 4.2.3 (R Core Team 2023). Using the Bray–Curtis dissimilarity index to calculate distance matrices relative to the taxa abundance group at the class level of taxonomy and CAZyme composition, a principal coordinate analysis (PCoA) was performed. To look for correlations between the metadata from the samples and the taxonomic diversity, a Mantel test of the abundance matrix versus metres below sea level (mbsl), latitude and longitude metadata of samples, and metres below sea floor (mbsf) were calculated. Data visualisation of PCOAs was performed using the vegan, pragma (Oksanen et al. 2017) and geosphere (Hijmans 2020) R packages. Linear discriminant analysis effect size analysis (LEfSe) of the taxonomic matrices of archaea and bacteria and were made in the Hutlab’s Galaxy tool (Segata et al. 2011) (LEfSe cutoff: Kruskal–Wallis Alfa value Kruskal–Wallis = 0.05, Alfa value Wilcoxon test = 0.05, LDA score > 3.0). A similar analysis was performed on the extracellular CAZyme matrix with an LDA score > 3.5.

### Construction of metagenomic assembled genome (MAG) and functional analysis

MAG were annotated, reconstructed, and refined using the Squeeze-Meta with pipeline v.1.4.0 (Tamames et al. 2019) (parameters: mode = sequential, assembly = extassembly, doublepass, lowmem). Genomic bins with low completeness (< 75%) and high contamination were removed (> 10%). Bins were refined with the remove_duplicate_markers.pl program of the SqueezeMeta pipeline. The taxonomic classification of these bins was performed by GTDB-Tk v2.1.0 (parameters: classify_wf) against the GTDB database v-207 (Chaumeil et al. 2020). CAZyme modules and Cazyme gene clusters (CGC) were annotated using dbCAN2 (Zhang et al. 2018); (hmmscan cutoffs: E-value < 1e-15, coverage > 0.35, DIAMOND cut-offs: E-value < 1e-102, Hotpep (Frequency > 2.6, Hits > 6), CGCFinder (Distance <  = 2, signature genes = CAZyme + TC). Marker genes (MG) were annotated with FetchMG v-1.2 (Kultima et al. 2012).

Normalisation of CAZyme counts between MAG was carried out using the equation: [(Number of the CAZyme module in the MAG/Number of the CAZyme module in the metagenome sample)/Median (MGs in metagenome sample)] × 10^6^. The list of MGs can be downloaded from the mOTU website https://motu-tool.org/fetchMG.html.

Soil MAG taxonomically assigned to Alphaproteobacteria, Gammaproteobacteria, and Bacteroidia classes (Nayfach et al. 2021) were randomly selected with the same criteria as our MAG (Completeness > 75% and Contamination < 10%). CAZyme modules and CAZyme gene clusters (CGC) were annotated using dbCAN2 (Zhang et al. 2018); (hmmscan cut-offs: E-value < 1e-15, coverage > 0.35, DIAMOND cut-offs: E-value < 1e-102, Hotpep (Frequency > 2.6, Hits > 6). The integrated matrices were written using R, bash, Perl, and Python and are available at http://github.com/Ales-ibt/Metagenomic-benchmark.

We used the Phylophlan 3.0 pipeline to calculate the phylogeny of the reconstructed MAGs, as well as the soil MAGs, using amino acid sequences (Asnicar et al. 2020). We used the Phylophlan database (Segata et al. 2013) that includes 400 universal marker genes and Diamond v0.9.24.125 (Buchfink et al. 2021) to map the database against our proteomes. Multiple-sequence alignments (MSA) were performed with MUSCLE v3.8.31 (Edgar 2004), and the trimAl v1.4.rev22 software (Capella-Gutiérrez et al. 2009) for the trimming of gappy regions. Finally, for the calculation and refinement of the trees, we used the Maximum likelihood estimation with the software IQ-TREE v2.0.6 (Nguyen et al. 2015) and RaxML v.8 (Stamatakis 2006), respectively, with 100 bootstraps. The tree representation was made using the Interactive Tree of Life (iTOL) Version 6.8.1. (2023). Retrieved from https://itol.embl.de/ (Letunic and Bork 2021).

## Results and discussion

### Correlations of metagenome samples based on available metadata

We analysed 37 metagenomes from all over the world. The physicochemical variables of most of our sediment samples were not available for comparison. However, they all come from shallow sediments at the interface with the water column, for which metadata such as geographic parameters (latitude and longitude, depth in metres below the seal level (mbsl), and depth in metres below the seafloor (mbsl) are known. Most of the samples retrieved were shallow coastal samples (Fig. 1).

Twenty samples were taken below 1000 mbsl and 17 above (Fig. 1 and Supplementary Table 1). This allowed us to test the correlation between environmental variables and the abundance diversity matrix at the class level.

We calculated a Mantel test to test whether the structure of the taxonomic community was correlated with geographical and spatial parameters (Supplementary Table 2). Our results showed a significant and positive correlation between depth (mbsl) and taxonomic diversity (Bray–Curtis dissimilarity matrix), while geographical distances and sediment depth (mbsf) were not significant (Supplementary Table 2). To further analyse this positive correlation between depth of the water column and taxonomic diversity, we performed a linear regression of both dissimilatory matrices (Supplementary Fig. S1). A low R-squared value (0.0404) suggests that depth below sea level does not explain much of the variation in taxonomic dissimilarity. Although there is a correlation between a greater depth of the water column and thin sediments, because the amount of organic matter is depleted and oxygen penetration is found throughout the sediment a trait that would make substantial differences in the microbial populations, many of our samples were taken in the first centimetres (from 0 to 2.23 mbsf) where the community utilises oxygen. (D’Hondt et al. 2015).

For that, we decided to make a metagenomic profile of the samples based on the taxonomical diversity of the community against its metabolic potential. Given the fact that only a sample did measure oxygen and understanding that all our shallow samples are a gradient between the oxic and anoxic layers of the sediments, we decided that the best way to get a comparison would be to see which respiratory metabolism prevails in each sample. This doesn’t recreate the geochemical conditions of each sample, but it does make a fine approach to understanding the community structure of marine sediments.

**Fig. 2:**
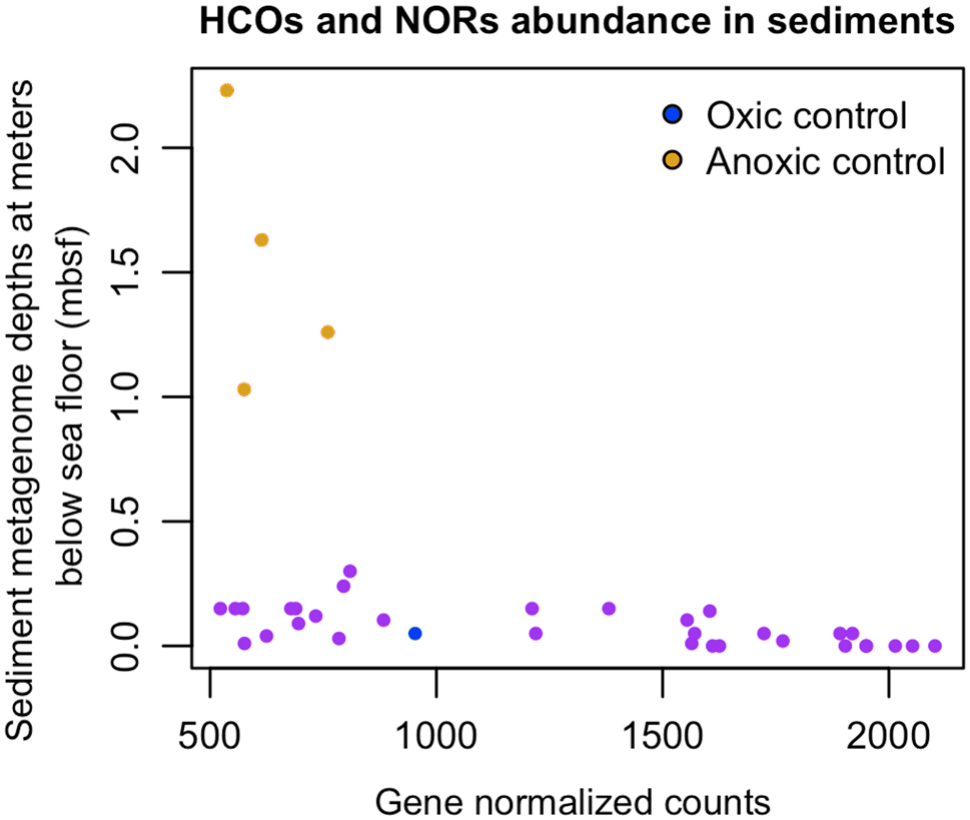
Dispersion graph of HCOs and NORs classified in each metagenome with the HCO database. The x-axis shows normalised gene counts of the reads, and the y-axis is the depth in metres below the sea floor of every metagenome sample (mbsf). Colour code; metagenome samples = purple; oxic control = blue; anoxic control = yellow. Graph made with the ggplot2 package of R

To this end, we assign categories to our samples (oxic/anoxic environments) based on the basis of their gene content of heme-copper oxygen reductases (HCO) and nitric oxide reductases (NOR). HCOs and NORs are enzymes found in the last complexes of many respiratory chains in microorganisms (Sousa et al. 2011). As reference, we used four sediments found in Loki’s Castle labelled as anoxic and one from the South Pacific Gyre labelled as oxic and which also has physicochemical measurements of oxygen (Supplementary Table 1). Anything greater than the normalised counts of HCOs and NORs in the oxic control was considered oxic and everything below was considered anoxic. Our results show 18 metagenome samples that can be considered oxic and 13 anoxic. Some of the samples assigned to the oxic label were shallow samples (under 1000 mbsl) and although there is a correlation between a greater depth of the water column and thin sediments, considering that the amount of organic matter is depleted and oxygen penetration is found throughout the sediment, many of our samples were taken in the first centimetres (from 0 to 2.23 mbsf) where the community utilises oxygen. (D’Hondt et al. 2015). This could be the reason why these samples below 1000 m below sea level are above the oxic control. (Fig. 2, Supplementary Table 1).

**Fig. 3:**
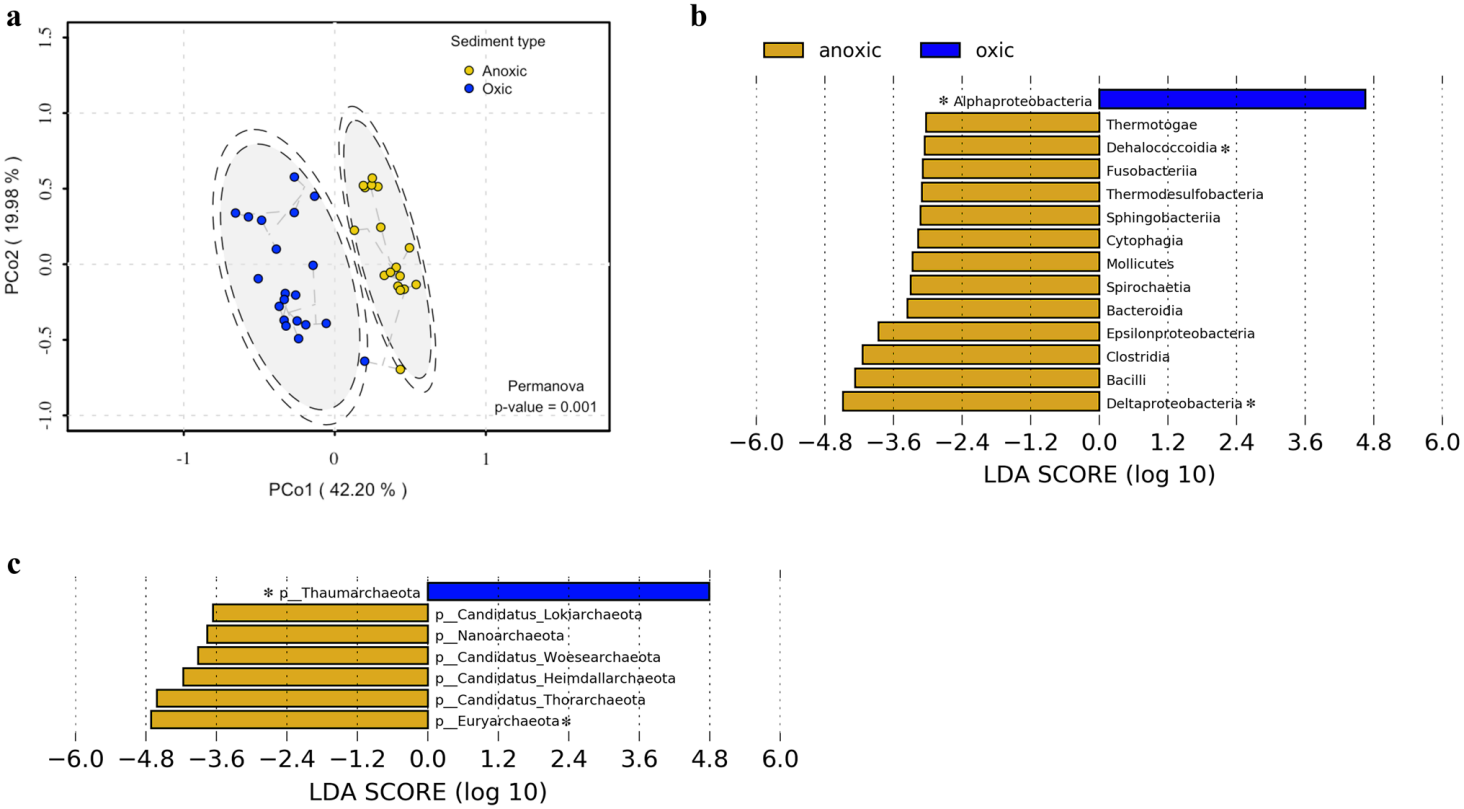
**a** Principal coordinate analysis (PCoA) of a Bray–Curtis dissimilarity matrix of taxa at the class level of sediment samples. The colour code indicates to which category of metadata they belong (blue = oxic; yellow = anoxic). Graph created with the vegan package R. **b** linear effect size discriminant analysis (LEfSe) to identify significant taxa between samples with the 'anoxic' and 'oxic' classes of bacteria and phylum in the case of Archaea. Taxonomic groups show LDA > 3.0 values with p < 0.1. The effect of size and power of statistical analysis was calculated with alfa values of 0.5 and 0.5 for Kruskal–Wallis (classes) and Wilcoxon (subclasses), respectively. Taxa with ‘*’ are reported as those under oxic or anoxic conditions (Orsi et al. 2018; Hoshino et al. 2020; Raggi et al. 2020)

Once we established the abundance of HCO and NOR as a condition in the samples, a principal coordinate analysis (PCoA) based on the relative taxonomic abundance at the class level (Bray–Curtis dissimilarity matrix) showed a clear separation of the samples labelled oxic and anoxic (62.18% of the variance explained in CoA1 and CoA2) (Fig. 3a, Supplementary Table 3). Samples were clustered into two groups; In the oxic group, samples from the deep Gulf of Mexico (Godoy-Lozano et al. 2018; Zhao et al. 2020) are reported without hydrocarbon or methane seeps (Zhao et al. 2020). The South Pacific Gyre is the only sample with an oxic level and an oligotrophic layer (Tully and Heidelberg 2016). Samples from Korea and Antarctica present anthropogenic disturbances; the Korea metagenomes are beach samples, the Davis Station are shallow samples rich in nutrients, and oxygen is consumed in the first centimetres of the sediment (Leeming et al. 2015). In the anoxic group, samples from the Gulf of Mexico (Delaware University), the Basin and Loki’s Castle, the Hydrate Ridge of the Pacific, and the Santa Monica Mounds were clustered together. These have been reported to have seepages of hydrocarbons or related compounds (Zhao et al. 2020), hydrothermal vents with anaerobic metabolism (Jaeschke et al. 2012; Kauffman et al. 2018; Bäckström et al. 2019), and mud volcanoes (Kauffman et al. 2018; Bäckström et al. 2019) (Fig. 3a).

Once we saw a clear separation between labels, we explored differences in taxonomic composition between the oxic and anoxic samples through a LEfSe analysis (Segata et al. 2011) based on bacteria and archaea abundance matrices at the class and phylum levels, respectively (Fig. 3b, Supplementary Figs. S2 and S3). LEfSe provides biomarkers based on different metadata categories (in this case oxic and anoxic traits).

The oxic samples showed an enrichment in Alphaproteobacteria. However, anoxic samples were enriched in several bacterial classes: Epsilonbacteria, Deltaproteobacteria, Bacilli, Clostridia, Fusobacteriia, Dehalococcoidia, Bacteroidia, Sphingobacteriia, Cytophagia and Thermodesulfobacteria. Among the Archaea phyla, Thaumarchaeota are significantly enriched in oxic samples, while Candidatus Bathyarchaeota, Euryarchaeota, and Candidatus Lokiarchaeota are indicators of anoxic samples. This is consistent with the literature where it is known that anoxic sediments are enriched with strictly anaerobic groups such as sulphate-reducing bacteria of the Chloroflexota phylum and Deltaproteobacteria and methanogenic archaea, such as Euryarchaeota, while in oxic sediments there is prevalence of the Alphaproteobacteria class in bacteria and Thaumarchaeota phylum in archaea (Biddle et al. 2008; Orsi 2018; Hoshino et al. 2020). Our results found that the classes Dehalococcoidia and Deltaproteobacteria of the Chloroflexota phylum along with other anaerobic classes such as Clostridia, Thermodesulfobacteria, Fusobacteriia bacteria and Euryarchaeota archaea were indicative of an anoxic environment, while the Alphaproteobacteria class of bacteria and Thaumarchaeota archaea (Tully and Heidelberg 2016; Hoshino et al. 2020) were indicative of oxic samples (Fig. 3b).

In summary, both groups exhibited significant differences in the classes of bacteria and the archaea diversity that appear to match the anoxic/oxic conditions of the microorganisms reported in marine sediments, as well as the genes reported (Fig. 3b).

### CAZyme profile of marine sediments

**Fig. 4:**
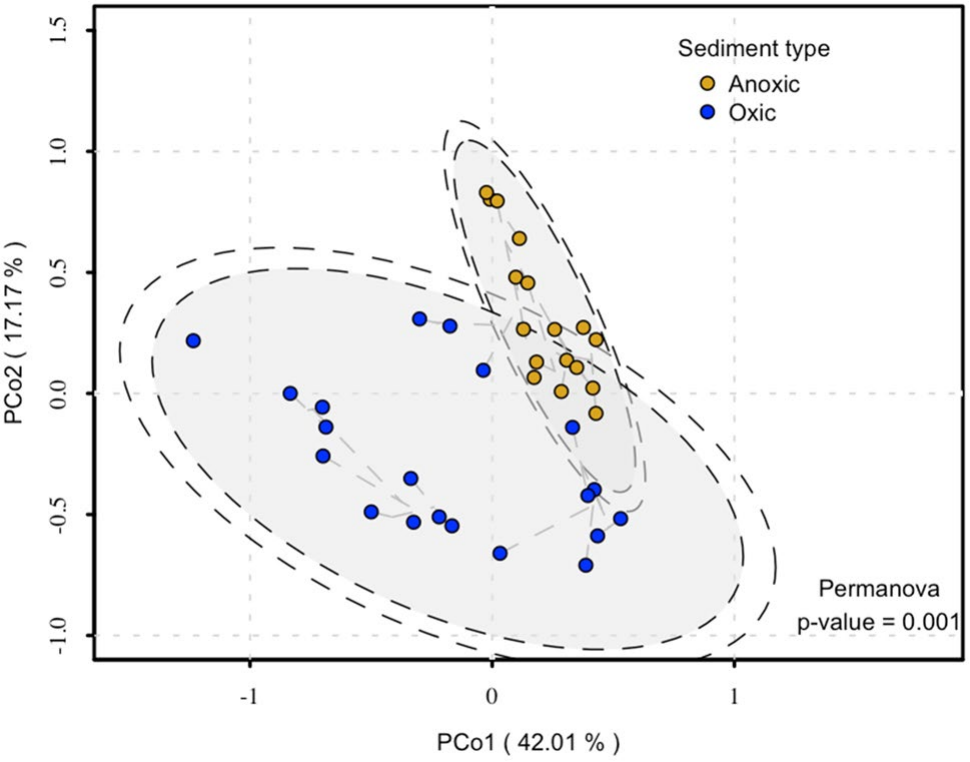
Principal coordinate analysis (PCoA) of a Bray–Curtis dissimilarity matrix from the CAZyme module normalised read counts of our sediment samples. The colour code indicates to which category of metadata they belong (blue = oxic; yellow = anoxic). Graph created with the vegan package R

We examined the distribution of carbohydrate-active enzymes (CAZyme) content within the metagenomes. To accomplish this, we performed a principal coordinate analysis (PCoA) using normalised counts of all CAZyme modules identified within each metagenome sample. Like our findings on beta diversity, our samples showed separation between oxic and anoxic conditions (59.18% of the variance explained in CoA1 and CoA2) (Fig. 4).

**Fig. 5:**
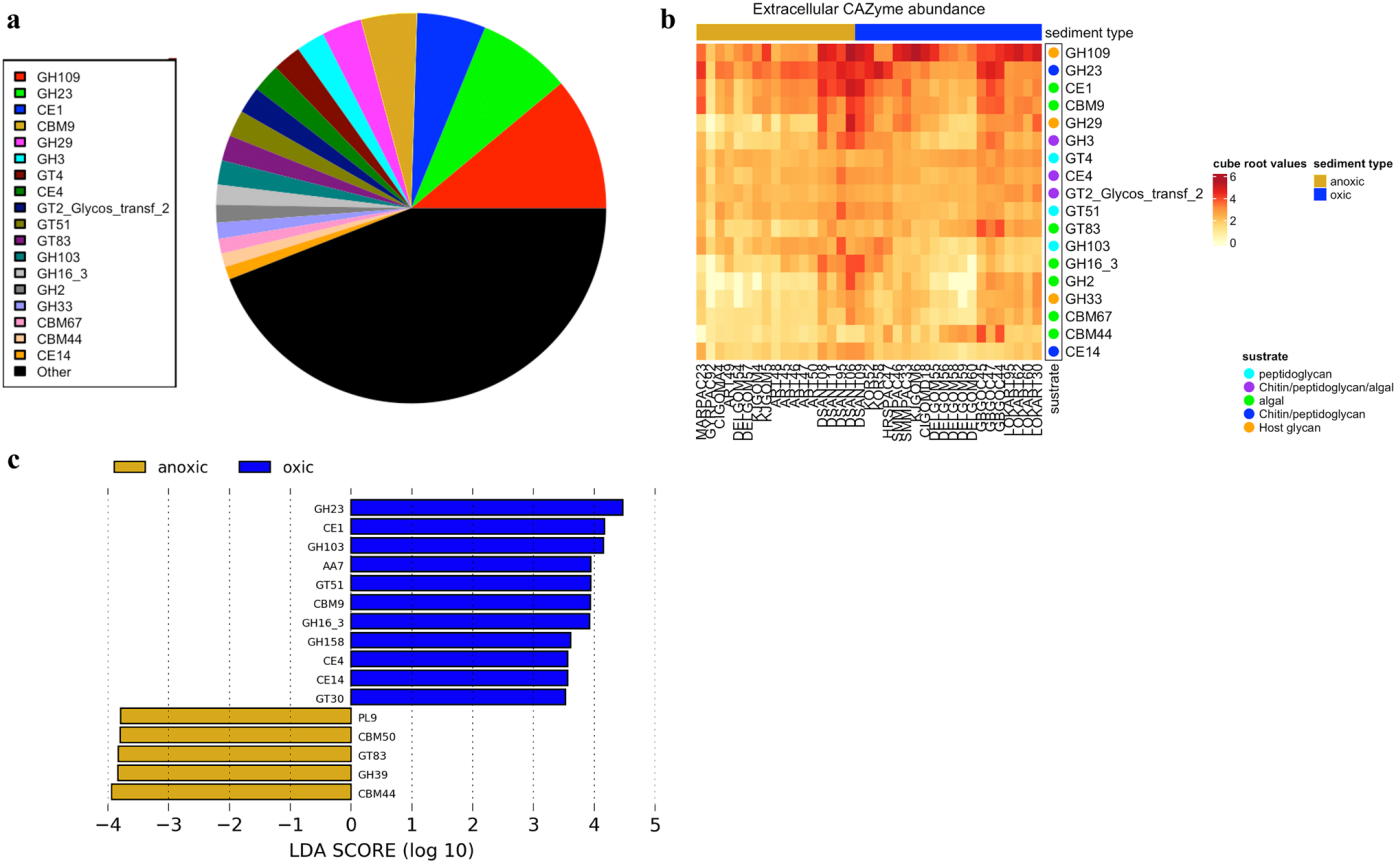
**a** Pie chart of the most abundant modules annotated for all sediment samples. **b** Heatmap of the abundance of extracellular CAZyme modules in sediment samples. Carbohydrate binding modules (CBMs), carbohydrate esterases (CEs), glycoside hydrolases (GHs) and glycoside transferases (GTs). The colour code of the modules refers to the substrate to which the modules are targeted reported in the literature (Lombard et al. 2013; Orsi et al. 2018). The column side colour represents the metadata label (yellow = anoxic; blue = oxic). **c** Linear effect size discriminant analysis (LEfSe) to identify significant extracellular CAZyme modules between samples with the ‘oxic’ and ‘anoxic’ classes. Auxiliary activities (AAs), carbohydrate binding modules (CBMs), carbohydrate esterases (CEs), glycoside hydrolases (GHs) and glycoside transferases (GTs) and polysaccharide lyases (PLs). The CAZyme groups show LDA > 3.5 values with p < 0.1. The effect of size and power of the statistical analysis was calculated with alfa values 25 of 0.5 and 0.5 for Kruskal–Wallis (classes) and Wilcoxon (subclasses), respectively

Given the assumption that carbon turnover in marine sediments is carried out by microbial organisms that use secreted enzymes to store carbon over time (Orsi et al. 2018), we decided to search for extracellular CAZymes. We performed a functional annotation of CAZyme modules that had a peptide signal against the CAZyme database (Lombard et al. 2013). We categorized sequences into the six classes of the CAZy database, which are implicated in the creation, breakdown, and identification of carbohydrates. These classes are glycoside transferases (GTs), glycoside hydrolases (GHs), carbohydrate esterases (CEs), carbohydrate binding modules (CBMs), polysaccharide lyases (PLs), and auxiliary activities (AAs). Eighteen extracellular CAZyme modules were found in quantities higher than 1% of all total CAZyme annotations (accounting for 55.94% of all CAZymes annotated in our metagenome samples). Of these modules, GH109, GH23, and CE1 were the most abundant (Fig. 5a). Their abundance was particularly high in the following metagenomes: Guaymas Basin (GBGOC), Davis Station from Antarctica (DSANT), Korean beaches (KOR), South Pacific Hydrate Ridge (HRSPAC47), Loki’s Castle (LOKART) from the Arctic, Santa Monica Mounds (SMMPAC), and the Gulf of Mexico (CIGOMD18 and KJGOM6) (Fig. 5b).

The metagenomes had an extracellular inventory of CAZyme, primarily targeting algal and necromass detritus (see Fig. 5b). Among the prevalent modules engaged in the breakdown of algal debris were glycoside hydrolase modules GH2, GH3, and GH16_3, as well as carbohydrate esterase CE1. The binding modules included CBM9, CBM44, and CBM67.These modules are composed of enzyme families with β-galactosidases, β-glucuronidases, β-mannosidases, exo-β-glucosaminidases activities in the case of GH2 and GH3, where glycoside hydrolases and phosphorylases perform a wide range of functions that involve biomass degradation and remodelling of plant and bacterial cell walls. GH16_3 breaks laminarase, a carbohydrate found in brown algae (Qin et al. 2017) while CE1 has acetylxylan esterases (EC 3.1.1.72), feruloyl esterases (EC 3.1.1.73) activities, and many other esterases such as PHB depolymerases. CBM9 and CBM44 are modules targeting cellulose binding domains mainly xylan and other carbohydrates cellulose binding domains and CBM67 targets binding to L-rhamnose, a carbohydrate produced by microalgae (0–13.3 of algal composition%) (Brown 1991) (Fig. 5b) (Lombard et al. 2014).

For necromass degradation, the GH23 and GH103 modules contain families of peptidoglycan lytic transglycosylases. GH23 has also been found to have chitinase activity. Furthermore, known activities of the CE4 and CE14 families include enzymes such as acetylxylan esterases, chitin deacetylases, chitooligosaccharide deacetylases, and peptidoglycan deacetylases (CE4) and diacetylchitobiose deacetylase (EC 3.5.1.-) chitin disaccharide deacetylases (CE14). (Lombard et al. 2014). Finally, for host glycan degradation, the GH29 module contains α-L-fucosidases, and the GH109 modules conform to -N-acetylgalactosaminidase, α-N-acetylgalactosaminidase, and β-N-acetylhexosaminidase. GH33 sialidase or neuraminidase (EC 3.2.1.18) targets the sialic acid of the host glycan (Fig. 5b).

It is documented that bacterial communities dominate shallow sediments, which are primarily composed of clay, cellular envelopes of planktonic organisms, and organic matter (Bienhold et al. 2016). Genes related to the degradation of recalcitrant carbon, including cellulose, chitin, or peptidoglycan, are expected to play an important role in marine sediments (Tully and Heidelberg 2016; Bradley et al. 2018; Orsi et al. 2018). Necromass contributes significantly to meeting the energy demand of up to 13% of the microbial community in shallow sediments when it is oxidised under oxic or anoxic conditions. The oxidation of one cell per year can provide sufficient energy to support the demand of thousands of cells in sediments with low energy resources, potentially positioning necromass oxidation as a primary carbon source for microorganisms unable to survive in energy-poor environments (Bradley et al. 2018). The fact that mineralization and adsorption of biopolymers in sediment particles could reduce the accessibility of other carbohydrates (Orsi et al. 2018) this could make cell envelopes, such as peptidoglycan, a preferred choice for secreted CAZyme modules found to be the most abundant (Fig. 5a). Most of these CAZyme modules are found across a broad spectrum of life forms but are concentrated in bacteria (Lombard et al. 2014).

Some of the most abundant modules differed between the oxic and anoxic samples. The CAZyme modules GH23, CBM9, GH16_3, GT51, CE4, and CE14 were significantly more abundant in oxic samples. On the other hand, CBM44 and GT83 were found to be different in relation to anoxic samples. Interestingly, despite quite opposite distributions, both CBM44 and CBM9 can bind cellulose (Fig. 5c).

### Reconstruction of MAG and their potential to degrade carbohydrates found in marine sediments

To better understand the community involved in carbohydrate turnover in marine sediments, we recovered MAGs from each metagenome sample. Here, we present 494 metagenome-assembled genomes (MAGs) reconstructed from the 37 metagenomes, each of which represents a snapshot of the microbial communities sampled from different sediments. Almost two-thirds of genomes are substantially complete with a completeness < 80% and a contamination < 10%, while the rest have a completeness < 75% and contamination < 10%. MAG sizes range from 0.75 to 9.56 Mbps. MAGs are distributed throughout the phylogenetic tree and cluster into 443 bacterial MAGs and 51 archaeal MAGs comprised in 103 and 3 class-level taxonomic groups, respectively, with 360 MAGs taxonomically assigned to the species level based on 95% average nucleotide identity. Most of them belong to the classes of Proteobacteria phyla (Gammaproteobacteria and Alphaproteobacteria) and Bacteroidea. (Supplementary Fig. S4; Supplementary Table 4). 

**Fig. 6:**
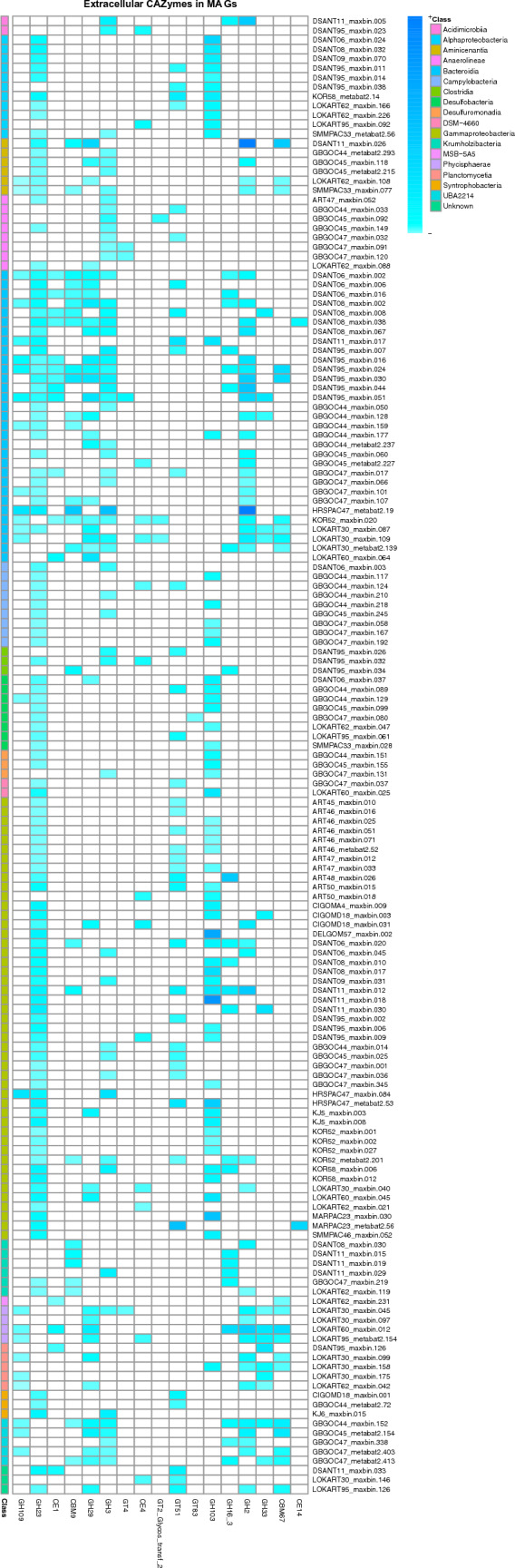
Heatmap of the most abundant extracellular carbohydrate-activated enzyme (CAZymes) modules found in our metagenome-assembled genomes (MAG) samples. The side-colour label is for the taxonomy at class level annotated to the MAG and it is read from top to bottom. For visualisation purposes, MAG that did not have a minimum of the main CAZyme Modules were discarded from the figure. (Refer to the Github repository for full gene counts encoding CAZymes in full MAG)

We selected the 18 CAZyme most abundant modules found in our annotations of the metagenome samples and searched and annotated them in the MAGs to see if they are responsible for the carbohydrate turnover found in the metagenome as a whole. We concentrate on secreted CAZymes modules and CAZymes modules corresponding to CAZyme Gene Clusters (CGC) (Fig. 6 Supplementary Tables 5, 6). We highlight the modules involved in the degradation of necromass and algal debris as they play the most important role in marine sediments as shown before (Orsi 2018),

The GH23 module was the most abundant in sediment MAG and no CBM44 modules were found in any of the MAGs. MAGs from the classes Alphaproteobacteria, Bacteroidia, and Gammaproteobacteria had more than one module of extracellular CAZymes (Fig. 6). Alphaproteobacterial MAGs had GH103 and GH23 modules. The Alphaproteobacterial MAGs belong to the Rhodobacteraceae and Methyloligellaceae families with species found in marine environments, including *Pseudorhodobacter*, *Sulfitobacter*, *Roseicyclus* and *Hyphomicrobium* (Uchino et al. 2002; Rathgeber et al. 2005; Yoon et al. 2007; Vuilleumier et al. 2011) and other species of the genus *Methyloceanibacter*, which had been previously reported in North Sea sediments (Vekeman et al. 2016) (Supplementary Tables 4, 5).

The Bacteroidia MAGs contained CAZyme modules in at least one MAG except for module GT83; CAZyme composition of the main modules found in our metagenome samples (GH109, GH23 and CBM9) was higher in the family Flavobacteriaceae were MAG assigned to the genus *Prevotella* (DSANT95_maxbin.044), Maribacter (DSANT06_maxbin.002, DSANT06_maxbin.016 and DSANT95_maxbin.030) *Pricia* (DSANT95_maxbin.051, DSANT95_maxbin.024 and DSANT95_maxbin.016) *Eudoraea* (DSANT11_maxbin.017), Aureibaculum (DSANT06_maxbin.006 and DSANT08_maxbin.008) along with other abundant modules (Fig. 6; Supplementary Tables 4, 5).

The species *Prevotella*, *Maribacter*, and *Aureibaculum* had been recovered from marine sediments from the Pacific Ocean and Yellow Sea (Reed et al. 2002; Nedashkovskaya et al. 2004; Zhao et al. 2019). *The Pricia* genus had previously been isolated from a sample of sandy intertidal sediment collected from the Antarctic coast (Yu et al. 2012) which is consistent with the place it was recovered (Davis Station). *Eudoraea* species were isolated from coastal waters of the Adriatic Sea (Alain et al. 2008).

Finally, MAGs without GH23 modules such as the classes of Phycisphaerae, UBA2214, Planctomycetia, and Bacteroidia contained GH109, GH2, GH29 and CBM67. UBA2214 was also enriched with GH3 modules. MAG from UBA2214, Phycisphaerae, and Planctomycetia MAG were assigned to the Zgenome-0027, Anaerohalosphaeraceae and Thermoguttaceae families, respectively. Species from these families are found in marine sediments and low oxygen aquatic environments (Dedysh et al. 2020; Pradel et al. 2020; Chiciudean et al. 2022). Furthermore, four Bacteroidia MAG assigned to the Bacteroidales order showed a similar CAZyme inventory (Fig. 6, Supplementary Table 5).

As CAZymes are also known to work in conjunction with other CAZymes and proteins forming CGCs, we decided to search for clusters involving the main CAZyme modules found in our metagenomes.

In general, CGCs targeting the GH23 module often came attached to a CBM50 module; GH3 module often came attached to a CBM6 module and GH2 modules often came with CBM67 modules (Supplementary Table 6). CBM50, a module for the recognition of chitin or peptidoglycan (Ohnuma et al. 2008), has already been abundantly reported in marine sediments (Orsi et al. 2018). CBM6 is known for the recognition of xylanases, lichenases, β-agarases, laminarinases and deacetylases, and CBM67 is known for the recognition of rhamnose, both carbohydrates are found in algal content (Lombard et al. 2014).

MAG belonging to Bacteroidia and Gammaproteobacteria have the highest number of CGCs. Bacteroidia MAG classified as *Prevotella* (DSANT95_maxbin.044) and Gammaproteobacteria *GCA-001735895 sp009937625* (KOR58_maxbin 012.fasta. contigs.refined) had the highest number of CGCs of all, with five including GH3, GH23 and GH2 modules in the case of Prevotella and targeting GH23, GH103, GT51 and CE4 modules. (Supplementary Table 4, Supplementary Table 5, Supplementary Table 6). The Alphaproteobacteria MAG contained CGCs targeting GH23, GH103, and GH3 modules. Gammaproteobacteria CGCs were found targeting CE4, GH103, GT51, CBM9, GH23 and GH3 modules.

Even though assembled MAG cannot cover all sediment diversity, we did find a group of MAGs annotated to classes that were abundant in our samples, such as Bacteroidia, Alphaproteobacteria, and Gammaproteobacteria. We did find the CAZyme inventory and CGCs that contained the most abundant modules found in our metagenomes in these classes of bacteria. Furthermore, the MAGs of Bacteroidia, Alphaproteobacteria and Gammaproteobacteria found having important CAZyme modules belong to genera or families found or isolated in marine environments, making these classes some of the main drivers for carbohydrate transformation in marine sediments. It is well known that the Bacteroidota phylum is considered the primary phylum for carbohydrate degradation (Lapébie, et al. 2019). All our MAGs from this phylum belonged to Bacteroidia. Phylum Proteobacteria was the most prevalent one in sediment samples. Most of the taxa we found belong to Gamma and Alpha Proteobacteria (47.75–13.88% and 33.83–6.91% of relative abundance, respectively) (Supplementary Table 7; Supplementary Figs. S2, S4).

We successfully identified and analysed the MAGs from metagenome samples (Supplementary Figs. S4; Supplementary Table 4), shedding light on the key players in carbohydrate turnover in marine sediments. These classes showed the presence of the most abundant CAZyme modules and CAZyme gene clusters (CGCs) that correspond to carbohydrate degradation in marine environments. The presence of these CAZymes and CGCs in marine-derived MAG indicates their critical role in carbohydrate transformation in marine sediments.

This highlights the importance of the Bacteroidota phylum in carbohydrate degradation, particularly the Bacteroidia class, and the significant contributions of both Gamma and Alpha Proteobacteria to the observed taxa in marine sediment samples.

### CAZyme profile of marine sediment taxa vs. soil sediments

Since Alphaproteobacteria, Gammaproteobacteria, and Bacteroidia had such a rich inventory of CAZyme for carbohydrates found in marine sediments, we decided to explore how different were the CAZyme inventories of our MAG to those of MAG of Alphaproteobacteria, Bacteroidia, and Gammaproteobacteria selected from soil samples published by Nayfach et al., (2021) using the same selection criteria (Completeness > 75% Contamination < 10%).

Marine sediments and soil are rich ecosystems of microorganisms and are crucial components of the Earth's surface, as they can sequestrate carbon and play a role in carbon recycling. (Arndt et al. 2013; Bardgett and Putten 2014).

MAGs of these classes were mainly from different families compared to the sediment MAG we recovered which clustered together in a phylogenetic tree (Supplementary Fig. S5); Gammaproteobacteria MAGs found in sediments group together across all in different clusters; the first group comprised sulphide oxidising bacteria from the family Beggiatoaceae, bacteria that lives in surficial sediments and sediment–water interfaces (Teske and Salman 2014); the second group clustered bacteria mainly from the acidiferrobacterales which has uncultivated genera that perform dark carbon fixation in coastal sediments (Dyksma et al. 2016); The third group gathers bacteria from six different orders mainly from two families (UBA4575 and SZUA-229) whose sequences have been mainly found in marine environments (Parks et al. 2022); cluster four has bacteria from the order Pseudomonadales with comprises different families of microorganisms found in marine environments such as Moraxellaceae, Halomonadaceae, HTCC2089 and Halieaceae (Park et al. 2012; Matsuyama et al. 2015; Qiu et al. 2021); cluster five are bacteria mainly from the family Woeseiaceae (order Woeseiales) who has been found in marine sediments (Hoffman et al. 2020); and finally cluster 7 has bacteria from the family Nitrosomonadaceae which comprise a group of ammonia oxidiser bacteria and has been found in marine environments (Prosser et al. 2014). In Alphaproteobacteria there are two clusters, one of bacteria that belongs to the Rhodobacteraceae family and another one of the order Rhizobiales (families Hyphomicrobiaceae and Methyloligellaceae). The MAGs of Bacteroidia from sediment also cluster together mainly in two groups one of the order Bacteroidales and the other one of the family Flavobacteriaceae, all of them with species isolated from marine environments (Nedashkovskaya et al. 2004) as discussed. There is also a singleton that belongs to the MAG of the Prevotella species sp018054505 which shows a greater genetic divergence. As mentioned above, this MAG was found to have the most CGCs in relation to the most common CAZyme modules, and the fact that Prevotella species have been found in marine environments due to anthropogenic contamination of sewers near the Norwegian Bore beach (Bagi and Skogerbø 2022) just like in the Davis Station marine sediment samples (where this MAG was reconstructed) could indicate more extensive evolutionary changes in this particular MAG. The fact that sediment MAGs from the same taxonomic class cluster together suggests that these microorganisms often possess shared ecological adaptations. In the literature has been suggested that cluster distributions could be interpreted as evidence of habitat filtering where a group of closely related species often share a trait that allows them to persist in a given habitat (Horner-Devine and Bohannan 2006). These adaptations could include responses to environmental stressors, such as oligotrophy conditions in marine sediments. The marine sediment communities adapt to low-energy conditions and are selected to survive under these conditions. Although mutation rates are low, recombination could affect sediment microorganisms and cause variations in its gene content (Orsi 2018). Over time, this can result in the observed clustering in the phylogenetic tree. (Supplementary Fig. S5; Supplementary Table 8).

**Fig. 7:**
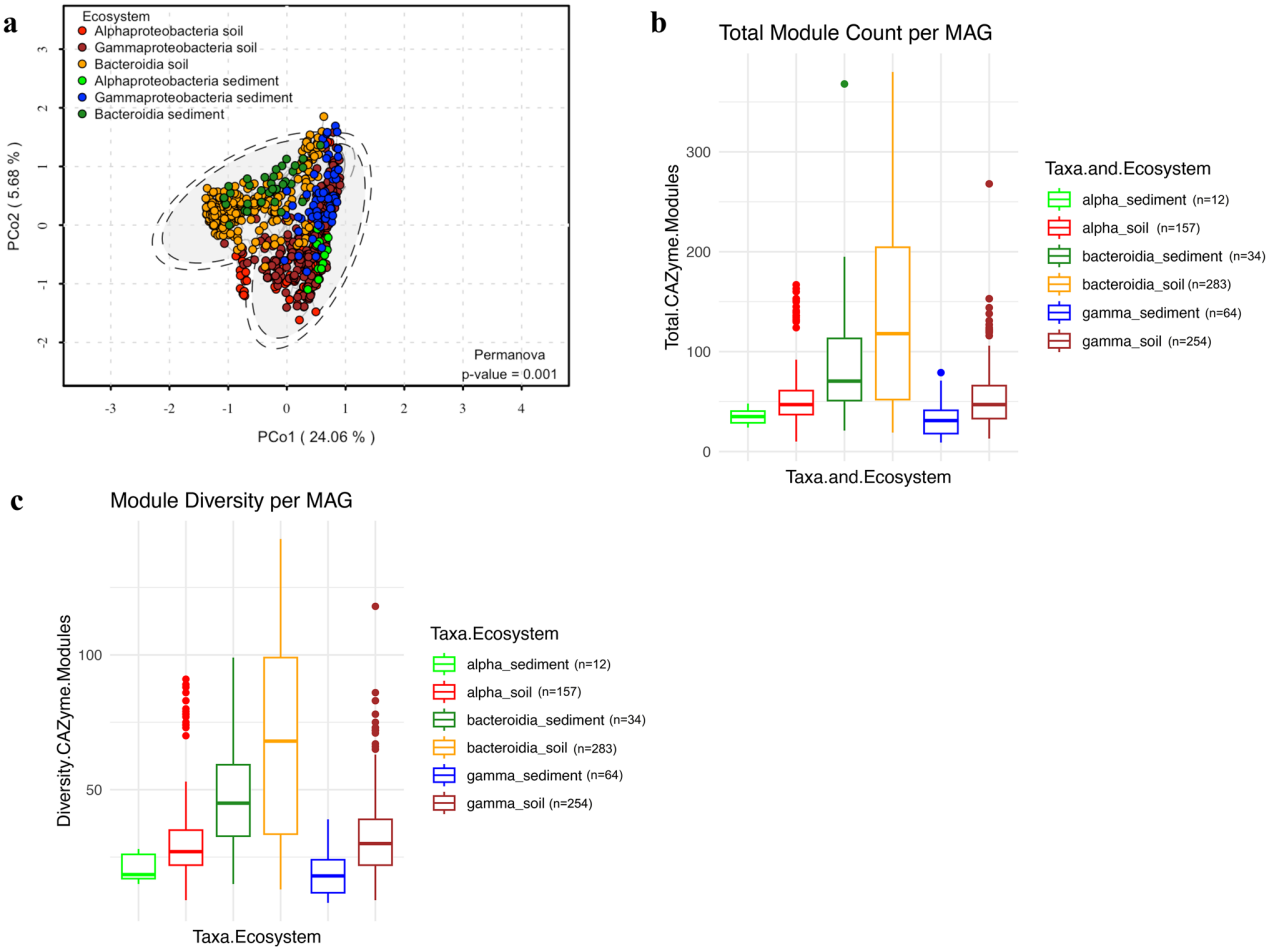
**a** Principal coordinate analysis** (**PCoA) based on CAZymes identified within soil and sediment MAG. The occurrence of habitat and taxonomy of each MAG is colour coded. The number of MAG is in parentheses. The counts of the CAZyme modules were normalised to percentages to construct a Bray–Curtis dissimilatory matrix. **b** Boxplot showing the total CAZy gene count per MAG (CAZyme modules abundance) within type of class and habitat (soil vs. sediment). **c** Boxplot CAZyme functional diversity (number of CAZyme modules per MAG) within type of class and habitat (soil versus sediment). All CAZymes classes of the CAZy database (Lombard et al. 2013) classification were considered. The box plots show the median values and the lower and upper quartiles

To see how different these MAGs were in terms of CAZyme repertoire, PCoA analysis of the counts of all CAZyme modules found in each MAG showed that the composition of CAZyme appeared to be similar between the phyla where Alfa and Gamma Proteobacteria clustered together, as did Bacteroidia MAG (29.74% of the variance explained in CoA1 and CoA2) (Fig. 7a).

The main difference between the classes recovered from the MAG of sediments compared to the MAG of soil was the number of CAZyme modules found between them and the diversity of the CAZyme modules: all classes of soil MAG had a total number of modules greater (Fig. 7b) and more diverse (Fig. 7c) compared to those we recovered from marine sediments where the Bacteroidia class was the one that had more counts and more diverse CAZyme modules. This is consistent with studies of MAG in environments where CAZyme modules are phylogenetically conserved, among microbial phyla, but some specificity toward habitat is present where soil is an ecosystem where richness in and diversity in CAZyme modules has been found in contrast to marine environments such as marine sediments (López-Mondéjar et al. 2022). Furthermore, the Bacteroidetes phylum to which the Bacteroidia class belongs has been reported as the main class for carbohydrate transformation, as it uses a large inventory of CAZyme (Lapébie et al. 2019).

This comparison between the MAG of marine sediment and soil metagenome-assembled genomes of Alphaproteobacteria, Gammaproteobacteria, and Bacteroidia reveals interesting differences that highlight the contrasting ecological roles and environmental pressures these bacteria experience in their respective habitats. The higher number and diversity of CAZyme modules found in soil MAG compared to marine sediment MAG support the idea that soil microbial communities are exposed to a wider variety of organic substrates, including plant biomass, animal detritus, and complex soil organic matter. This diversity of substrates likely drives the need for a broader suite of enzymatic capabilities in soil microorganisms, as reflected in their CAZyme repertoire.

On the contrary, marine sediment environments may be more homogeneous in terms of organic substrate availability, possibly due to the predominance of marine-derived organic matter, such as phytoplankton and other marine organisms. This could explain why marine sediment MAG possess a less diverse CAZyme profile compared to soil MAG.

Another possible explanation could be that the marine sediment environment is more energy-limited compared to the soil, leading to selective pressure for organisms that can efficiently degrade available organic matter with a smaller set of enzymes. This could potentially lead to a more streamlined CAZyme profile in marine sediment bacteria.

Despite these differences, the fact that Alphaproteobacteria, Gammaproteobacteria, and Bacteroidia from soil and marine sediments cluster together in the PCoA analysis suggests a core set of CAZyme modules that are conserved within these taxonomic groups, likely reflecting shared evolutionary histories and core metabolic functions.

This study underscores the importance of considering the ecological context when studying the functional capabilities of microbial communities. The stark differences in the CAZyme profiles between soil and marine sediment bacteria underscore how environmental factors can shape the functional potential of microbial communities. Therefore, it is essential to take these factors into account when studying the ecology and function of microorganisms in different environments.

## Conclusion

In this study, we classified 37 metagenomes from around the world with few physicochemical metadata by comparing the community’s potential to use oxygen as the last electron acceptor as a marker to classify them as oxic or anoxic. We find a clear difference between our sediment samples in terms of taxonomy and CAZyme content in the context of this classification. We established a profile of the most abundant extracellular CAZymes in our samples, where 18 CAZyme modules were abundant in all and were found to primarily target carbohydrates from necromass degradation and algae detritus, which is consistent with the environmental conditions found in sediments. We find significantly different modules targeting the same substrate depending on oxic and anoxic conditions. Most of the main abundant CAZyme modules that we found were of bacterial origin.

Finally, we recovered MAG from the samples, which were assigned to the classes Alphaproteobacteria, Gammaproteobacteria, and Bacteroidia. The MAG contained extracellular modules of the main CAZymes that were also annotated in our metagenomes, as well as CGCs that had those modules as part of the CAZyme machinery. Module GH23, which targets peptidoglycan and chitin substrates, was found in almost all our MAG. The MAG that did not contain the GH23 module had other different main modules that target host glycan and plant detritus. These taxa are the bacteria that mainly drive carbohydrate transformation in marine sediments, although further studies are needed to fully confirm this. Our findings provide valuable information on the community structure and function of carbohydrate turnover in marine sediments, highlighting the key roles that specific bacterial classes play and their associated CAZyme inventories and CGCs.

It is important to note that many of the MAG we reconstructed belonged to taxa already found in marine environments and that the classes Bacteroidia, Alpha, and Gammaproteobacteria are found in our metagenomes in abundance. When we compare the MAG from sediments with other MAG from the same taxa assembled from soil samples, we find a similar profile, as expected from taxonomy, but with fewer total and diverse CAZyme modules in sediment MAG. This is a response to the oligotrophic conditions in marine sediments, in contrast to soil conditions. Although the MAGs from our samples give us a glimpse of the microbial community, the number of recovered MAGs is not sufficient to encapsulate the great diversity of the microbial community of marine sediments. Furthermore, it is necessary to study deep subsurface sediments to better understand the CAZyme inventory compared to shallow marine sediments.

### Supplementary Information

Below is the link to the electronic supplementary material.

Supplementary file1 (DOCX 13530 kb)

Supplementary file2 (XLSX 538 kb)

## Data Availability

The authors confirm that all supporting data, code, and protocols have been provided within the article or through supplementary data files. Metagenomes from Illumina sequenced shotgun sediment samples were obtained from NCBI (https://www.ncbi.nlm.nih.gov/genome) and from the Instituto de Biotecnología de la UNAM. All details and accession numbers from the samples can be found in the Supplementary Material and GitHub repository: https://github.com/RafaelLopez-Sanchez/marine_sediments

## Abbreviations

AA: Auxiliary activities
CE: Carbohydrate esterase
CBM: Carbohydrate binding module
GH: Glycoside hydrolase
GT: Glycosyltransferase
CAZymes: Carbohydrate-active enzymes
CGC: CAZyme gene cluster
MAG: Metagenomic assembly genomes
Mbsl: Meters below sea level
Mbsf: Meters below sea floor
PL: Polysaccharide lyase
