# Supplementary material for: Metagenomic analysis of carbohydrate-active enzymes and their contribution to marine sediment biodiversity
Source: World J Microbiol Biotechnol. 2024 Feb 13;40(3):95. doi: 10.1007/s11274-024-03884-5 (PMC10864421; doi:10.1007/s11274-024-03884-5)
Supplement: Supplementary file 1 — Supplementary file1 (DOCX 13530 kb) [file 11274_2024_3884_MOESM1_ESM.docx]

**Fig. S1.** Lineal regression of the Mantels test in abundance vs. mbsl dissimilatory matrixes.

**Fig. S2.** Relative abundance of bacteria from sediment samples at the taxonomic class level. Other is any class that does not have at least 1% abundance in one of the metagenome samples.

**Fig. S3.** Relative abundance of archaea from sediment samples at the taxonomic phylum level. Other is any phylum that does not have at least 1% abundance in one of the metagenome samples.

**Fig. S4.** Phylogenetic tree of the MAGs reconstructed in marine sediments. The classes taxonomic from the 10 most abundant MAGs reconstructed are colour coded (Refer to the Github repository for full taxonomic annotations in MAG). Concentric circles show A) The taxonomic class B) The completeness (%), C) The size of the MAG (Mbps). Taxonomy classes were annotated using GTDB database v-207 (Chaumeil et al., 2020). Bootstrap values in branches are coloured. Tree representation was made Interactive Tree Of Life (iTOL) Version 6.8.1. (2023). Retrieved from <https://itol.embl.de/>

**Fig. S5.** Phylogenetic tree of the MAGs assigned to Alphaproteobacteria, Gammaproteobacteria and Bacteroidia MAGs from soil and sediments. Concentric circles show A) The taxonomic class with the environment B) The completeness (%), C) The size of the MAG (Mbps). Taxonomy classes were annotated using GTDB database v-207 (Chaumeil et al., 2020). Dotted lines delimit the clusters of the marine sediment MAGs in the Lowest Common Ancestor they grouped (labels of family or order). Bootstrap values in branches are coloured. Tree representation was made Interactive Tree Of Life (iTOL) Version 6.8.1. (2023). Retrieved from https://itol.embl.de/

Supplementary Figures

Fig. S1


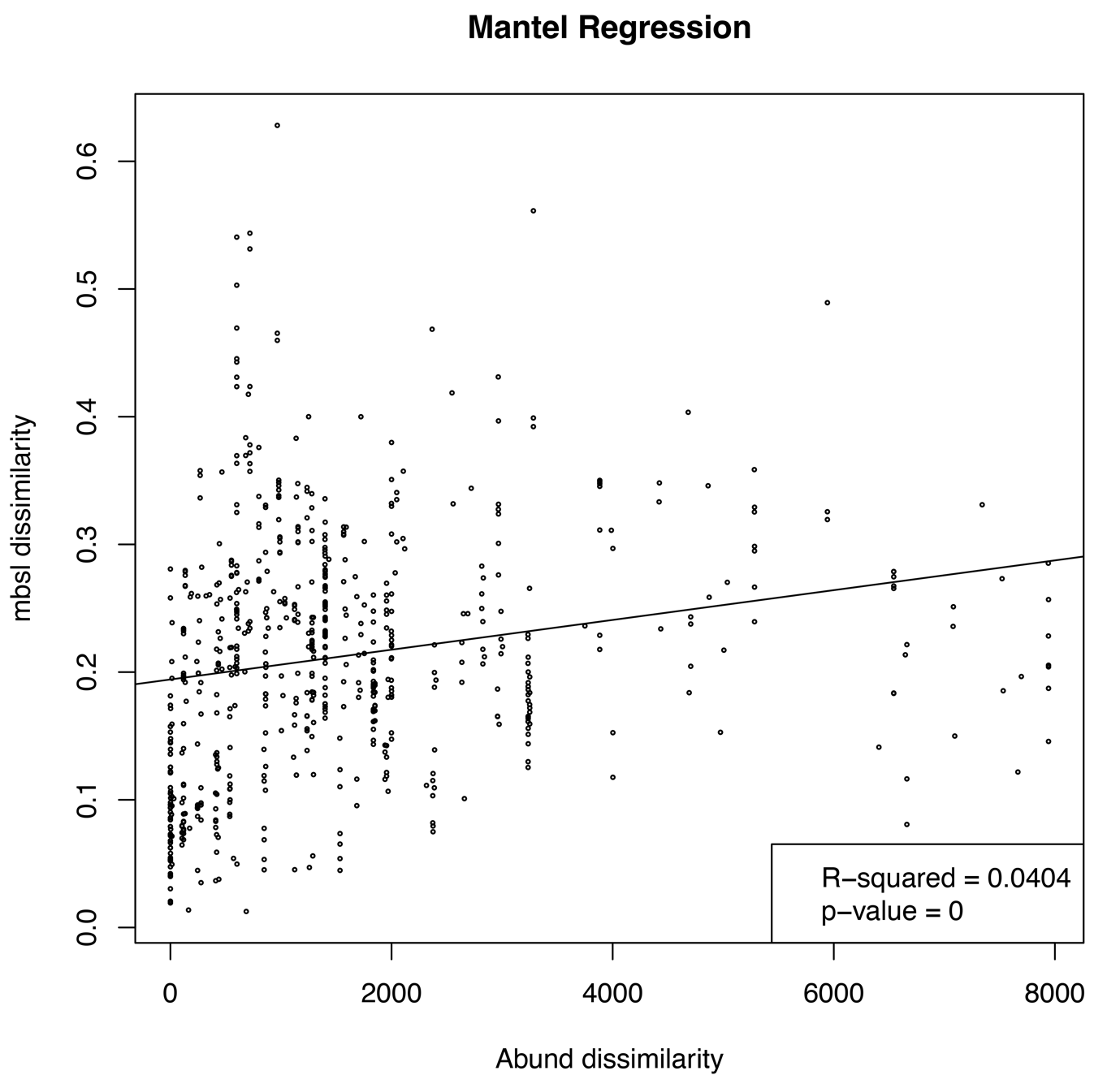


Fig. S2


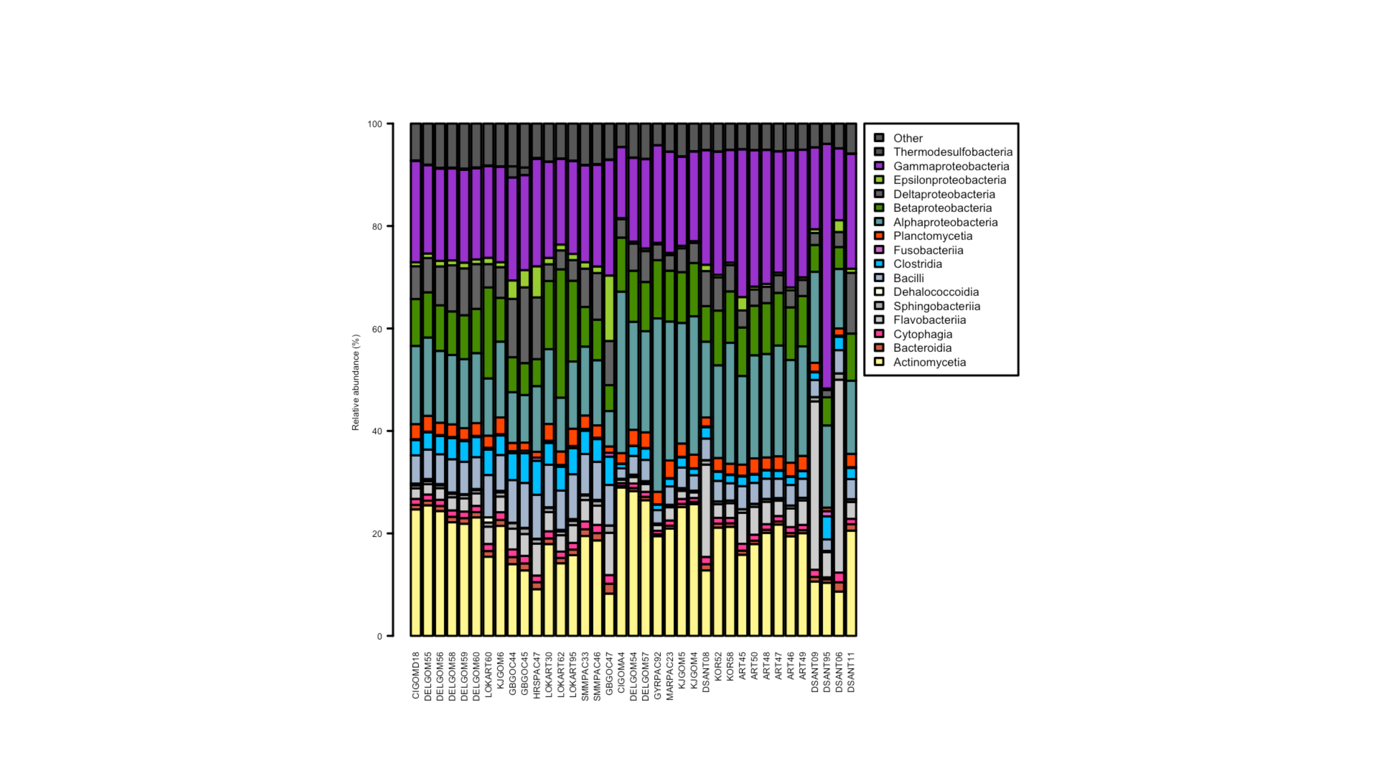


Fig. S3


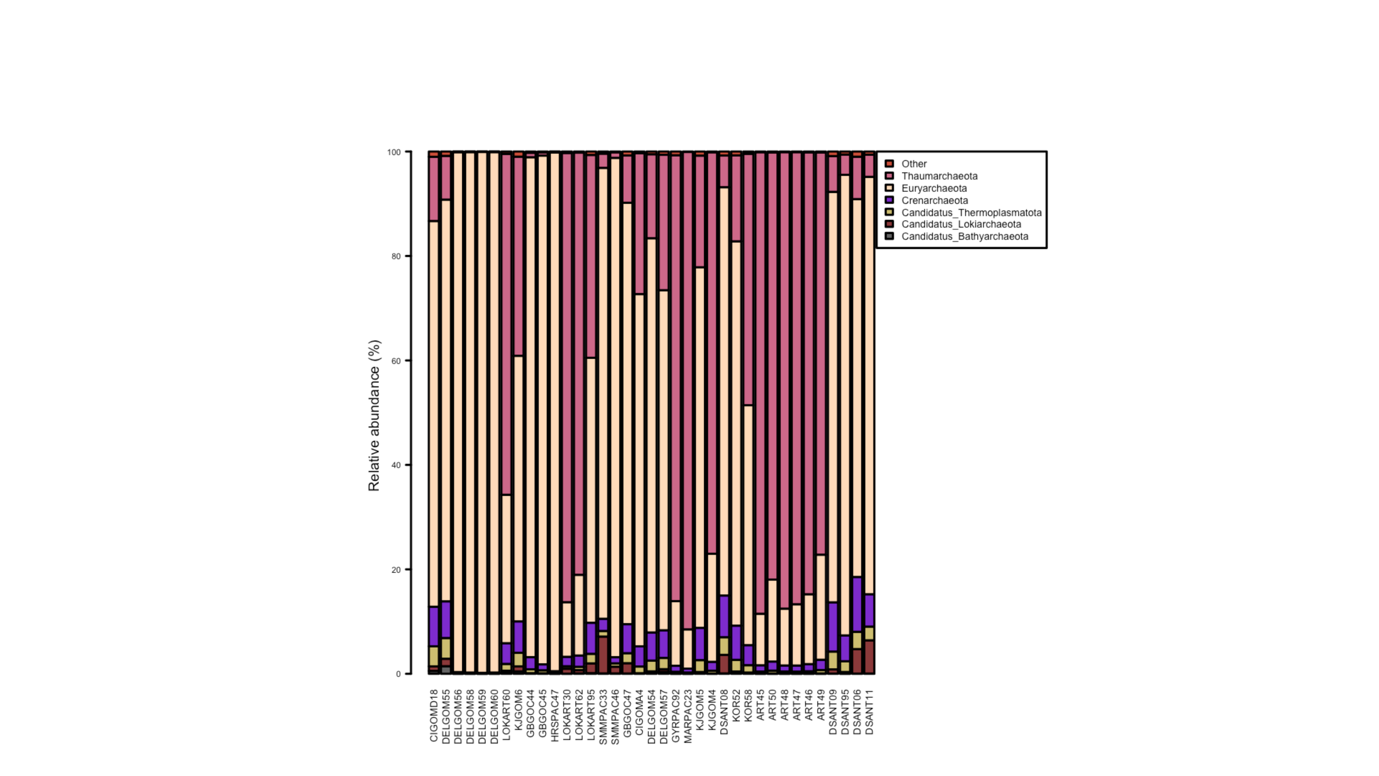


Fig. S4

Fig. S5
